# Reinfection of farm dogs following praziquantel treatment in an endemic region of cystic echinococcosis in southeastern Iran

**DOI:** 10.1371/journal.pntd.0011939

**Published:** 2024-03-27

**Authors:** Mehdi Borhani, Mohammad Ali Mohammadi, Mahbod Entezami, Mohammad Reza Baneshi, Saeid Nasibi, Joaquin M. Prada, Majid Fasihi Harandi

**Affiliations:** 1 Research Center for Hydatid Disease in Iran, Kerman University of Medical Sciences, Kerman, Iran; 2 State Key Laboratory for Zoonotic Diseases, Key Laboratory of Zoonosis Research, Ministry of Education, Institute of Zoonosis, College of Veterinary Medicine, Jilin University, Changchun, China; 3 School of Veterinary Medicine, University of Surrey, Guildford, United Kingdom; 4 School of Public Health, The University of Queensland Faculty of Medicine, Herston, Australia; 5 Modeling in Health Research Center, Institute for Futures Studies in Health, Kerman University of Medical Sciences, Kerman, Iran; Pontificia Universidad Catolica de Chile, CHILE

## Abstract

Cystic Echinococcosis (CE) as a prevalent tapeworm infection of human and herbivorous animals worldwide, is caused by accidental ingestion of *Echinococcus granulosus* eggs excreted from infected dogs. CE is endemic in the Middle East and North Africa, and is considered as an important parasitic zoonosis in Iran. It is transmitted between dogs as the primary definitive host and different livestock species as the intermediate hosts. One of the most important measures for CE control is dog deworming with praziquantel. Due to the frequent reinfection of dogs, intensive deworming campaigns are critical for breaking CE transmission. Dog reinfection rate could be used as an indicator of the intensity of local CE transmission in endemic areas. However, our knowledge on the extent of reinfection in the endemic regions is poor. The purpose of the present study was to determine *E*. *granulosus* reinfection rate after praziquantel administration in a population of owned dogs in Kerman, Iran. A cohort of 150 owned dogs was recruited, with stool samples collected before praziquantel administration as a single oral dose of 5 mg/kg. The re-samplings of the owned dogs were performed at 2, 5 and 12 months following initial praziquantel administration. Stool samples were examined microscopically using Willis flotation method. Genomic DNA was extracted, and *E*. *granulosus sensu lato*-specific primers were used to PCR-amplify a 133-bp fragment of a repeat unit of the parasite genome. Survival analysis was performed using Kaplan-Meier method to calculate cumulative survival rates, which is used here to capture reinfection dynamics, and monthly incidence of infection, capturing also the spatial distribution of disease risk. Results of survival analysis showed 8, 12 and 17% total reinfection rates in 2, 5 and 12 months following initial praziquantel administration, respectively, indicating that 92, 88 and 83% of the dogs had no detectable infection in that same time periods. The monthly incidence of reinfection in total owned dog population was estimated at 1.5% (95% CI 1.0–2.1). The results showed that the prevalence of echinococcosis in owned dogs, using copro-PCR assay was 42.6%. However, using conventional microscopy, 8% of fecal samples were positive for taeniid eggs. Our results suggest that regular treatment of the dog population with praziquantel every 60 days is ideal, however the frequency of dog dosing faces major logistics and cost challenges, threatening the sustainability of control programs. Understanding the nature and extent of dog reinfection in the endemic areas is essential for successful implementation of control programs and understanding patterns of CE transmission.

## Author summary

Cystic echinococcosis (CE), caused by the small tapeworm of dogs, *Echinococcus granulosus*, is considered as a prevalent zoonotic infection of human and livestock worldwide. Dogs play a crucial role in the parasite life cycle, serving as definitive hosts for the tapeworm and contributing to the transmission of the disease to humans and other livestock.

Praziquantel (PZQ) dosing of dogs is proposed as one of the main elements of CE control programs. Frequent PZQ dosing is required because of reinfection of farm dogs following feeding offal to them and this presents major logistic and financial problems. The frequency of PZQ dosing is dependent on the rate of dog reinfection in each endemic region. We explored the significance of farm dogs reinfection in an endemic area in southeastern Iran, to provide better understanding of CE transmission and developing effective control programs.

We monitored a cohort of 150 dogs previously treated for cystic echinococcosis by praziquantel. We showed that the prevalence of echinococcosis in the farm dogs, using PCR assay was 42.6% before praziquantel administration. To our surprise, a significant proportion of these dogs, approximately 17%, experienced reinfection with *E*. *granulosus*, 12 months following initial praziquantel administration. This finding was both alarming and informative. Our study emphasized the importance of responsible dog ownership behavior and proper disposal of livestock offal in endemic areas.

## Introduction

Cystic Echinococcosis (CE), caused by the metacestodes of *Echinococcus granulosus sensu lato* (s.l.), is considered as a prevalent zoonotic disease of human and livestock worldwide. CE is transmitted between carnivorous and herbivorous mammals as definitive and intermediate hosts respectively [1]. The infection occurred following accidental ingestion of eggs excreted in dog feces and dispersed in the environment. CE infection is highly endemic, and widespread in many parts of the world, especially in specific rural settings where humans and animals live in close proximity [2].

CE is present in the Middle East and Central Asia and poses a potential threat to the human population. Iran is considered as an endemic focus of CE, where high prevalence rate of infection with various genotypes of *E*. *granulosus* have been demonstrated in dogs as well as different livestock [2–4]. CE is estimated to impose a substantial cost to the society, including losses associated with human surgical and other treatment costs and livestock production losses. In Iran the overall annual monetary burden of CE, has been estimated at US$232 million [5]. Various biotic and abiotic agents play a role in the endemicity of CE. In addition, behavioral and socio-economic factors including close contact with dogs, outdoor activities, contaminated food and water are important factors in CE transmission to humans. Livestock husbandry is a major part of Iran’s economy and many parts of the country are inhabited by farmers, as well as the settled, nomadic and seminomadic pastoral communities, practicing livestock husbandry and use of shepherd dogs [6]. Pastoral lifestyle involves farm slaughter, home slaughter and unregulated slaughter sites, that facilitates the access of farm dog as well as free-roaming dogs to the livestock offal. This is one of the key determinants of CE transmission in many endemic areas of the world including Iran. In-depth understanding of the epidemiology of human and animal echinococcosis is required for developing effective control programs.

According to the World Health Organization (WHO), monitoring of echinococcosis in animal populations is essential for disease control and prevention programs [7]. Praziquantel (PZQ) dosing of dogs, livestock vaccination, improving abattoirs infrastructure, control at-home slaughter, meat inspection and removing sheep offal from the environment, management of free roaming dog (FRD) population, registration of owned dogs, and public and professional education interventions are proposed as the main elements of CE control programs [8]. However, due to the diverse epidemiological conditions of echinococcosis and its inherent complexities a one-size-fits-all strategy cannot be successfully implemented for interrupting transmission. Overall, four phases are considered for CE control including planning phase, attack, consolidation and maintenance [8].

Planning is an important phase of control as it needs to explore prerequisites including intersectoral coordination, sustainable funding, community participation and advocacy. One of the key elements in CE control plans is the availability of accurate baseline information from human and animal surveillance data [7,9]. As part of the baseline information, one of the most important data needed about echinococcosis transmission, is the epidemiological data of echinococcosis in dogs, as the most important definitive host of the parasite. Generally, dogs can be infected through feeding on livestock offal remained after slaughter at home or at sub-standard abattoirs. Praziquantel dog dosing is a key measure for CE control in many endemic countries, however dogs can be reinfected sometime after PZQ administration. The rate of reinfection in dogs is an indicator of the intensity of CE transmission in endemic areas. The frequency of dog deworming is dependent on the rate of dog reinfection in endemic regions and this is a major factor in the planning of CE control programs.

Theoretically, continuous dog dosing campaigns ensure critical decrease in CE transmission. However, in this circumstance, frequent dog dosing in endemic regions is associated with major logistic and financial problems including cost of the drugs, costs related to the drug delivery, staff education. Certainly, reinfection studies are essential to address various questions on the epidemiology of echinococcosis in dogs, and to provide baseline data for CE control and prevention.

Several studies investigating reinfection of dogs have been conducted in CE endemic regions including northwestern province of Xinjiang in China, northern Kenya, northern Libya, southern Argentina, Tunisia, Morocco and Uruguay [10–16]. Our knowledge on reinfection in dogs is very limited in the Middle Eastern countries including Iran. Therefore, understanding the nature and extent of dog reinfection in the endemic areas is essential for successful implementation of control programs and understanding patterns of CE transmission [17]. Additionally, understanding the spatial heterogeneity of disease prevalence can be used to understand where intervention and control measures are needed most. Poor knowledge of this issue in endemic regions leads to the waste of time and energy in preventive measures against cystic echinococcosis.

The findings may further assist relevant authorities in conserving and developing integrated management practices related to human and animal health, achieving an appropriate control approach for CE.

The aim of this study was to determine the reinfection rate with *E*. *granulosus* in a population of owned dogs after PZQ administration in a natural setting. We also evaluated the prevalence of sampled areas using a spatial model to highlight regions with increased disease risk. The results of this study can add to our knowledge about the dynamics of CE transmission in endemic countries.

## Methods

### Ethics statement

This study was approved by the Ethical Review Committee of Kerman University of Medical Sciences and the Research Center for Hydatid Disease in Iran (RCHD), code No. IR.KMU.REC.1398.468. The study was performed between December 2019 and December 2020.

### Study location and sample collection

Kerman is located on a high margin of Dasht-e Lut (Lut Desert) in the south-central part of Iran and 1,755 m (5,758 ft) above sea level, with the average annual rainfall of 142 mm. Kerman has a continental climate, with hot summers and cold winters and is surrounded by mountains. Based on the previous data of livestock population, the provincial veterinary authority in Kerman was asked to give us 100 epidemiological units (farms) selected by random sampling. One hundred and fifty owned dogs were included in the present study, belonging to 83 farms in 51 GIS points in the region. The study location coordinates of 51 points where the samples were collected, are provided in the supporting information (S1 Data File).

The owners of the dogs participating in the survey were informed of the main research goals. All the dogs found in each selected farm were examined. The dogs included in this study were mostly working dogs as sheep and/or guard dogs. Dogs with severe health conditions and those dogs whose owners refused to participate were excluded from the study.

We had thoroughly explained study objectives to the dog owners, ensuring they understood the importance of the study and fully comprehended the significance of sample collection. The dogs involved in this study were mostly tied up throughout the day, with limited freedom to roam. They were allowed to be freed during the night to carry out their role of guarding and protecting the properties and livestock. In this case, the fresh stool sample was collected from where the dog was restrained. In other cases, if a dog was observed defecating, the fresh stool was located, and the sample was collected from the uppermost surface of the feces with the observance of the health tips. All samples were labeled with dog data including dog name and/or number, age and sex. The samples were immediately transferred and stored in -70°C freezer for at least two weeks. A questionnaire was prepared for collecting data on the age, sex, risk factors of dog and human infection, dog dosing history, dog food, roaming status, home slaughter, and offal disposal behavior.

### Parasitological study

Stool samples were collected before PZQ administration (step 0). In order to eliminate dog tapeworms, all dogs were received a single oral dose of 5 mg/kg PZQ under the supervision, according to the WHO/WOAH guidelines [7]. Dogs were classified in three categories according to the body mass, small (<10 kg), medium (10–25 Kg) and large (>25 Kg) [18] and the dosage was administered to each animal accordingly. Re-samplings of the owned dogs were performed at 2 (step I), 5 (step II) and 12 months (step III) after initial PZQ administration [13].

Flotation-based methods using different high-density solutions are currently the method of choice for detecting taeniid eggs in canine feces. Using Willis flotation method, as one of the well-known floatation techniques of stool examination, the stool samples were microscopically examined in the laboratory. Briefly: a) 2 gr of feces was placed in a mortar, b) 100 ml of saturated NaCl solution was added and mixed with the stool, c) the fecal suspension was gently poured through a tea strainer into a test tube until a convex meniscus formed at the top of the tube, d) a coverslip was placed carefully on the top of the tube and the tube was left for 20 minutes, e) the coverslip was watchfully lifted off and transferred on a clean slide. Finally, the slide was thoroughly examined using an optical microscope [19].

### Molecular study

Genomic DNA of all stool specimens were extracted, using ExGene Stool DNA mini kit (GeneAll, South Korea), according the manufacturer’s instructions. The primers, Eg1121a, 5′-GAATGCAAGCAGCAGATG-3′ (forward) and Eg1122a, 5′-GAGATGAGTGAGAAGGA GTG-3′ (reverse) were used for specific identification of *E*. *granulosus sensu lato*. This primer pair amplifies a 133-bp fragment of a repeat unit of the parasite genome with suitable sensitivity and specificity [20,21]. The PCR was carried out in 25 μl reaction volumes in FlexCycler (Analytik Jena, Germany) machine. The thermal profile included an initial denaturation at 94°C for 5 min; followed by 35 cycles, each of 30 s at 94°C, 45 s at 50°C and 35 s at 72°C; and a final extension of 10 min at 72°C. The amplification products were subjected to electrophoresis on 2% agarose gel in TAE buffer.

### Data analyses

Kaplan-Meier method was applied to calculate cumulative survival rates. For reinfected dogs, time was defined as the infection time. For non-infected dogs, the time of last microscopic examination was defined as the censored time. To calculate the incidence rate of infection, number of positive cases was divided by total dog-time of follow-up. Log rank and Chi-square tests were used to investigate the statistical differences in CE prevalence between male and female dogs. All statistical analyses were performed in the R package.

### Spatial analyses

A spatial analysis of the sampled locations was conducted using the INLA package [22] in R. Following the method from P. Moraga (2019) [23], an Stochastic Partial Differential Equations (SPDE) model was developed. The model predicts prevalence following a binomial distribution:

$$Y_{i}|P(x_{i})∼Binomial(N_{i},P(x_{i}))$$

With Y_i_ representing the number of positive samples taken, N_i_ the number of samples tested at and P(x_i_)prevalence at location x_i_.


$$\log it(P(x_{i}))=\beta_{0}+S(x_{i})$$

With β_0_ representing the intercept (i.e. the average prevalence of the region) and S(x_i_) is the spatial random effect which follows a zero-mean Gaussian process.

A triangulated mesh was built to cover the city of Kerman and the surrounding area, which the SPDE model was created upon. The prevalence is predicted onto a raster of 150 by 150 pixels over a 0.6° longitude by 0.6° latitude square region around the city of Kerman. A relative risk of each pixel is calculated by the following equation:

$$R(x_{i})=\frac{P(xi)}{\beta 0}$$

With R(xi) representing the relative risk at location x_i_. The model outputs were visualized using the R Leaflet package [24]. Full model description and code available at https://github.com/MabEntez/Dog-reinfection-spatial-model-in-kerman.git.

## Results

A total of 150 dogs were registered for determining *Echinococcus* infection during a year. The sex and age distribution of the dogs is demonstrated in Table 1. Before praziquantel administration taeniid eggs were found in 8% of dog samples using stool microscopy. However, using the more sensitive molecular methods, it was determined that 42.6% of the stool samples were infected with *Echinococcus*.

**Table 1:** Demographic data of the farm dogs and dog ownership behavior of the farm dog owners in Kerman, southeastern Iran.

Variable	Number (%)
**Sex**	150 (100)
Male	118 (78.7)
Female	32 (21.3)
**Age**	150 (100)
Pup/young	129 (86)
Adult/old	21 (14)
**Feeding dogs with livestock offal**	
Dogs fed	150 (100)
Dogs not fed	0 (0)
**Deworming of dogs with PZQ**	
Yes	0 (0)
No	150 (100%)
**Livestock laughter at home/farm**	
Yes	150 (100%)
No	0
**Dog roaming status**	
Freed to roam at night	150 (100%)
Not freed to roam at night	0

**Fig 1:**
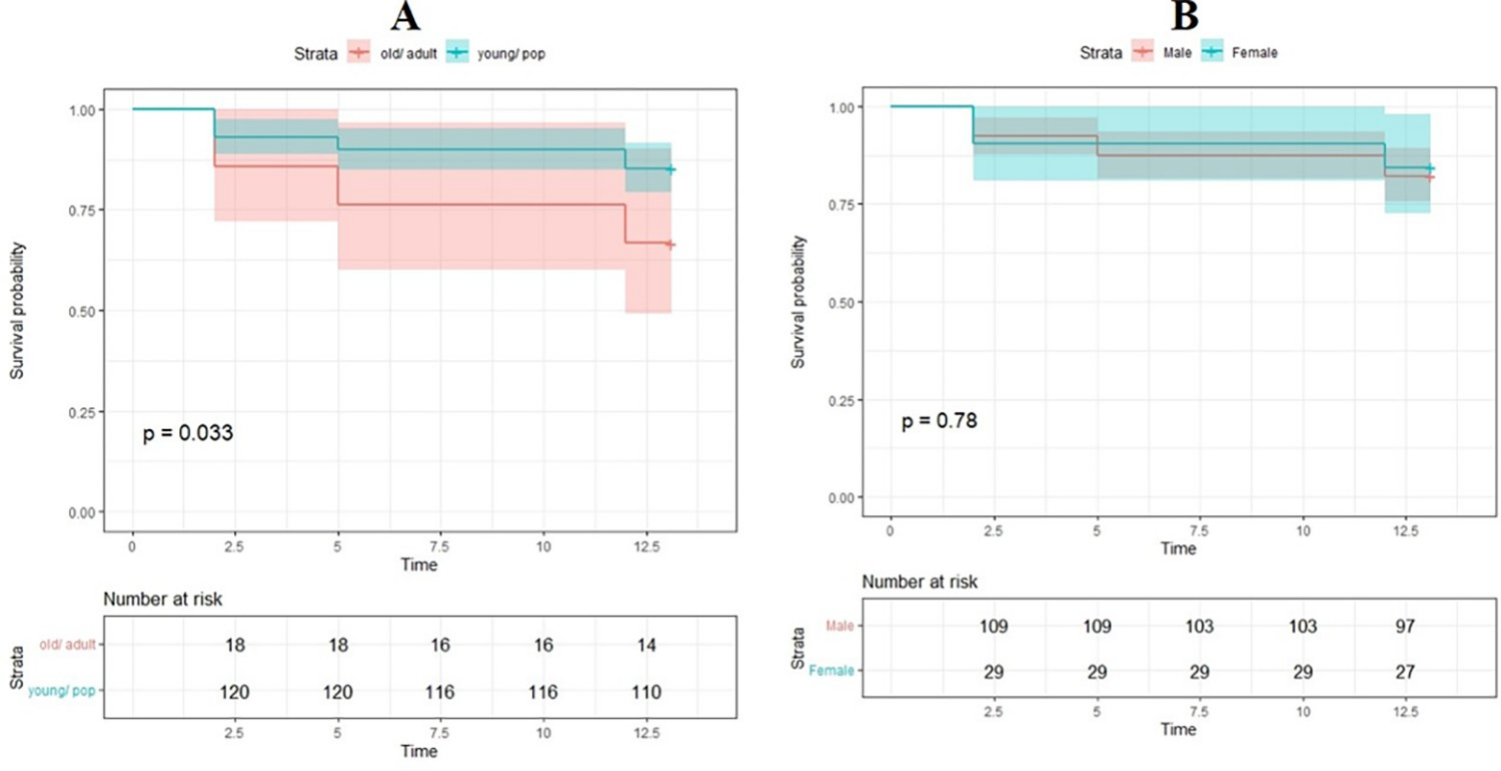
Kaplan-Meier Survival estimates among dogs reinfected with *Echinococcus granulosus*. Cumulative survival rate stratified by age (panel A) and sex (panel B).

A cohort of 150 owned dogs was registered to determine the reinfection rate with *E*. *granulosus* within the dog population. Results of survival analysis are shown in Table 2. Echinococcosis reinfection in different steps of the survey indicate 8, 12 and 17% reinfection rates in the steps I, two months post-treatment (mpt), II (5 mpt) and III (12 mpt), respectively (Fig 1). This means a survival rate of 92, 88 and 83% in the same time periods, i.e. no new event (new infection) was occurred in the dogs. The monthly incidence of reinfection in total owned dog population was 1.5% (95% CI 1.0–2.1, 26/1744). Findings showed that 8% of the dogs were found infected only after 2 months following PZQ dosing. Over a 12-month period a number of dogs were dropped out of the study due to the absence, loss, or death of the dogs at the study sites, so that at step I, 71% of the initial dog population (106 dogs) were sampled. At step II, 59% (88 dogs) and at step III, 60% of the initial owned dog population (90 dogs) were re-sampled.

**Table 2:** *Echinococcus granulosus* reinfection rate of farm dogs after 2, 5 and 12 months post treatment in Kerman, southeastern Iran.

Step (mpt*)	No. at risk	No. of events	Survival rate (CI)	Reinfection rate %
**I (2)**	150	12	92% (88%, 96%)	8
**II (5)**	138	6	88% (82%, 94%)	12
**III (12)**	132	8	83% (77%, 89%)	17

* months post treatment

There was a higher proportion of male to female dogs at a ratio of 3.6:1. The prevalence of *E*. *granulosus* between female and male owned dogs was not significant (P = 0.776). The monthly incidence of reinfection in male and female dogs was 1.5 and 1.3%, respectively. Dogs between the ages of 1 and 3 years were most common in the population. The monthly incidence of reinfection in pup/young dogs and adult/old dogs was found as 3.1 and 1.2%, respectively (p = 0.03) (Table 3). Dogs were mostly kept for the purpose of guarding the household and livestock. As shown in Table 1, all dog owners reported home slaughter and feeding dogs with livestock offal. Dog dosing with anthelminthic drugs was not reported by the dog owners.

**Table 3:** *Echinococcus granulosus* reinfection rate and monthly incidence of farm dogs after 2, 5 and 12 months post treatment in Kerman, southeastern Iran.

	Male		Female		Adult/old dogs		Pup/young dogs	
Step (mpt*)	**No at risk** (No. events)	**Survival rate** (CI)	**No at risk** (No. events)	**Survival** **Rate** (CI)	**No at risk** (No. events)	**Survival** **Rate** (CI)	**No at risk** (No. events)	**Survival rate** (CI)
**I (2)**	118 (9)	92% (88%, 96%)	32 (3)	91% (81%, 100%)	21 (3)	86% (70%, 100%)	129 (9)	93% (89%, 97%)
**II (5)**	109 (6)	87% (81%, 93%)	29 (0)	91% (81%, 100%)	18 (2)	76% (58%, 94%)	120(4)	90% (84%, 96%)
**III (12)**	103 (6)	82% (74%, 90%)	29 (2)	84% (72%, 96%)	16 (2)	67% (47%, 87%)	116 (6)	85% (79%, 91%)
**Monthly incidence**	1.5%		1.3%		3.1%**		1.2%**	

* months post treatment

** Statistical significance

**Fig 2:**
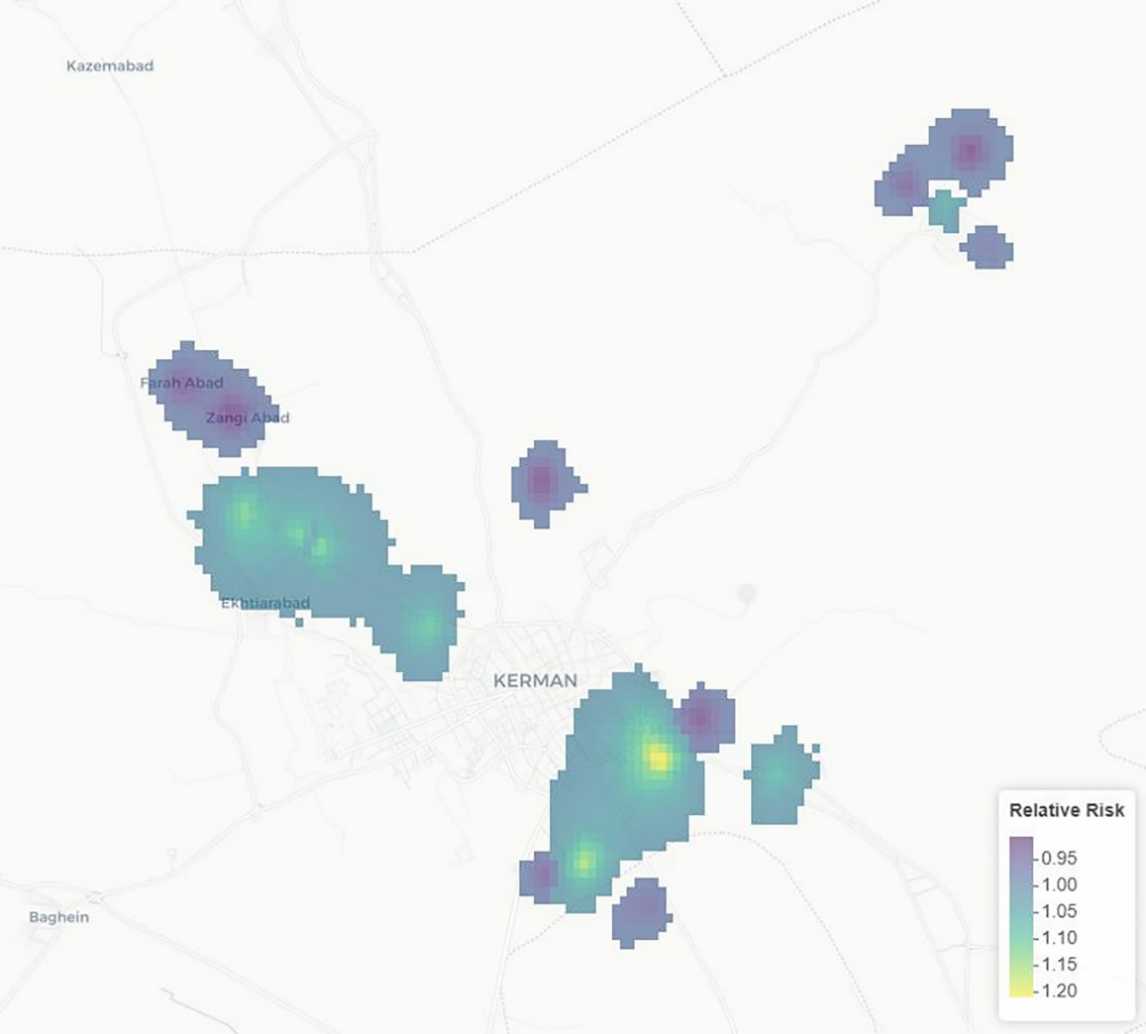
The relative risk of infection with *Echinococcus granulosus* predicted from sample data by the SPDE spatial model. Map data from OpenStreetMap. The map contains information from OpenStreetMap and OpenStreetMap Foundation, which is made available under the Open Database License. Data are available on GitHub: https://github.com/MabEntez/Dog-reinfection-spatial-model-in-kerman.git.

The main output of the SPDE spatial model is a relative risk of the sampled regions. For the sake of visualizing the outputs on the map, only pixels with a relative risk of less than 0.985 and more than 1.015 are presented on the map. This is shown in Fig 2. The relative risk ranged from 0.9 to 1.21, with the β_0_ (average prevalence in the population) at 0.415.

## Discussion

The present study investigated the reinfection status of owned dogs in an endemic region of cystic echinococcosis in Iran. CE is endemic in Iran and the prevalence of *Echinococcus granulosus* in dogs in different parts of Iran has been estimated between 6.8 and 55.7% [3]. PZQ dosing of dogs is the first line option for CE control programs, however many dogs got reinfected following PZQ treatment, and this presents a major challenge in the control programs. Reinfection rate of the owned dogs determines the optimum frequency of dog dosing [25]. Moreover, the rate of dog reinfection is one of the best indicators of the potential of CE transmission between humans and animals. Therefore, the knowledge about echinococcosis transmission among owned dogs can illustrate the extent to which dogs have access to livestock offal. The frequency of dog dosing is a major issue regarding the logistics, costs and sustainability of programs [2,13]. The SPDE model shows that there are distinct sample areas that show higher rates of infection, with a maximum increase of 20% in some areas. Areas with higher relative risk could be a sign of increased transmission or a neglect in control.

According to the WHO/WOAH, the efficacy of a single dose of oral PZQ (5mg/ kg) for the owned dogs is 99.9%; consequently, any test-positive dogs after drug intervention is considered as a new infection. Pre-patent period of *E*. *granulosus* in dogs is approximately 45 days. This is a sufficient period of time for echinococcosis to be established in the canine hosts [26]. Few studies have been performed in CE endemic countries to investigate *E*. *granulosus* reinfection rate of owned dogs. In the present study, a cohort of owned dogs in Kerman were followed for one year to evaluate dog reinfection status after the administration of a single dose of PZQ.

Findings of the study indicate that in a one-year period, no infection event has occurred in 83% of the dogs, this means an annual reinfection rate of 17% in the owned dogs. This is in accordance with the findings of a study on dog reinfection in Libya. A study in northwest Libya on owned dogs 15 months after praziquantel treatment estimated the reinfection rate of dogs as 22% [10] with a significant decrease in the prevalence from 21.6% to 9%. In northwest China, *E*. *granulosus* reinfection rate following praziquantel treatment after 3 months was 25% [11]. The corresponding number for the owned dogs in Argentina was 10% [14].

The prevalence of *E*. *granulosus* in rural dog population in Uruguay was 13.2%, and reinfection rates of dogs at 2, 4, 8 and 12 mpt were estimated at 0, 5.4, 18.6 and 27.9%, respectively [15]. In a cohort study in Tibetan communities of Sichuan, rate of reinfection of dogs at 2, 5 and 12 mpt was investigated. In this study the rate of canine *Echinococcus* coproantigen prevalence was reported from 21% in the baseline to 9.6%, two months post-treatment [13]. In three locations of the Middle Atlas, in Morocco, Amarir et al showed 23% of PZQ-treated dogs were re-infected 4 months following treatment. They concluded that 2 or 3 months interval between PZQ treatments in owned dogs controls the risk of reinfection of owned dogs and significantly reduces reinfection after the second treatment [27].

In a study in the northwestern Turkana district of Kenya, the natural infection rates with *E*. *granulosus* in dogs were assessed at 6, 12, 18, and 24-week intervals following PZQ. The prevalence in dogs were shown to reach to about 20% only six weeks following PZQ dosing. The prevalence of infection was finally returned to pretreatment levels within 24 weeks [16]. Comparing to our study, this reflects the significant differences in the epidemiology and infection pressure between the two regions, the Turkana region with one of the highest incidence rates of CE and Iran as a country with moderate incidence of CE.

In Tunisia the reinfection rate of rural semi-stray dogs with *E*. *granulosus* were examined following arecoline purgation at 2, 4, 8, and 12 months. Lahmar et al showed that reinfection with *E*. *granulosus* occurred very quickly after the end of PZQ treatment so that after 2 months the infection of dogs was not found significantly different compared to the day 0. Regular treatment of the dog population with PZQ every 60 days was recommended and it was suggested that dog dosing every 6 months, would decrease the pressure of *E*. *granulosus* infection in the rural regions of Tunisia [12]. Findings of the present study indicate a monthly incidence of 1.5%, this means the reinfection of nearly 10% of dog population in a 6-month period. However, it should be noted that the major part of reinfection has occurred in the first two months following PZQ treatment, i.e. 8% of dogs were quickly reinfected only after 2 months, followed by the remaining 8% reinfected after 10 months. It can be concluded that these dogs belong to the owners with irresponsible dog ownership behaviors such as regular feeding their dogs with livestock offal as well as practicing home slaughter. According to our field observations home slaughter and feeding dogs with livestock offal is a popular practice in the study area and all dog owners reported these practices at least once within last 12 months.

Cystic echinococcosis is a major zoonotic neglected tropical disease (NTD). Knowing *E*. *granulosus* reinfection rate of local dogs is an essential indicator of the potential of disease transmission in humans and livestock. In parallel with the findings of Lahmar et al in Tunisia and Moss et al in Sichuan, PZQ dosing of owned dogs every 2 months can significantly diminish dog infection and subsequent CE transmission in the endemic regions [12,13]. Therefore, continuous PZQ dosing at least 2–3 times a year greatly reduces the risk of human and livestock CE [28]. However, a practical and acceptable frequency of dog deworming in Iran have to balance the positive impact with operational sustainability and feasibility. Further field and modeling evidences are required to find the optimum dosing frequency with maximum effect on CE transmission. In addition, different reinfection patterns can be found in different endemic regions and in the case of Iran, where comprehensive studies on reinfection rates have been lacking, it is advisable to implement further studies in areas with different endemicity to understand the most suitable control strategy with regards to dog deworming.

It should be noted that some other factors can be of potential importance in determining the time interval of deworming campaigns. One major factor is the species/genotype of *E*. *granulosus* sensu lato. According to the studies performed in Kerman, both *E*. *granulosus* sensu stricto and *E*. *canadensis* G6 genotype have been reported from human patients in Kerman [29]. Developmental differences between species and genotypes of *E*. *granulosus* sensu lato, and variation in the pre-patent period in definitive host, is an important factor in the control programs [30]. It has been demonstrated that the pre-patent period is usually 6 weeks in dogs but it may range from 34 to 58 days depending on different genotypes of the parasite as well as the breed of dogs [31]. Therefore, a uniform anthelmintic deworming frequency cannot be proposed for all endemic areas and this frequency should be arranged according to several epidemiological data in each endemic region. In addition to the implementation of evidence-based deworming programs, EG95 livestock vaccination combined with effective science communication, public education and advocacy, can drastically reduce the incidence of CE in both humans and animals. Perhaps one of the biggest challenges in the endemic regions is the lack of knowledge about zoonotic diseases and their treatment, control and prevention. Increasing awareness through public education is one of the key measures for CE control in endemic communities.

At the same time some other measures to reduce the chance of dog reinfection are necessary including control of slaughter at home and standard meat inspection at official abattoirs. Another aspect of home slaughter is the traditional slaughter of livestock that is common in many cultures and religions. Livestock sacrifice is particularly prevalent during the largest Muslim religious festival known as Eid-al-Adha (Eid Ghorban, feast of sacrifice), where animals like sheep, goats, cattle and camels are sacrificed. It is essential to note that during this religious event, animals are slaughtered outside the abattoirs at home or during street festivals, and this is potentially contributing to the increased risk of zoonotic parasites transmission through increased chance of infected organs or carcasses to be available to the carnivores [6,32]. Unfortunately, the exact frequency of home slaughter practice in not known in many countries including Iran, however field observations in different parts of endemic areas indicate that this practice is prevailed throughout the country.

Several echinococcosis control programs have been implemented in some CE endemic regions, however there is no ongoing control program at national or sub-national levels in Iran [33]. Findings of a research project conducted in Kerman, Iran from 1991 to 1994 indicate a significant decline in the infection rate among dogs (dropping from 5% in 1993 to 1.5% in 1994) following culling of unowned free-roaming dogs and carrying out biennial PZQ treatment of the owned dogs [34]. Based on our findings, there is a need for a control program with a focus on both regular dog dosing with PZQ and EG95 livestock vaccination in Kerman. Regular PZQ dosing of dogs is central to a successful control program. However other CE control measures are needed, such as improving abattoirs infrastructure, control at-home slaughter and removing sheep offal from the environment and public and professional education intervention. Managing dog populations is a complementary approach for reducing burden of echinococcosis in combination with other control measures such as PZQ dosing, and targeting livestock.

Obviously, an effective CE control program requires consistent efforts and sustainable support. Control measures are facing serious logistic, financial and administrative constraints and need multi-/inter-sectoral coordination with a clear and comprehensive one-health approach linking knowledge and resources of veterinary and human medicine. An integrated management can be a solution to resolve the cost and logistic problems in the delivery of PZQ and EG95 vaccine. For instance, simultaneous EG95 vaccination of sheep and goat with enterotoxaemia vaccine can be considered as part of this integrated approach [9]. Preventing dog reinfection is essential for a successful control program, however there is no effective vaccine against echinococcosis in the definitive host. Vaccination of both the definitive and intermediate hosts is an ideal approach for achieving animal CE control targets. Introducing an effective vaccine against the adult forms of *E*. *granulosus* can be a great advantage to enhance other control measures, however no sound evidence is available and high quality studies are required to investigate the potential for an effective vaccine against canine echinococcosis. The lack of sufficient evidence of natural safety in the final host is one of the most important reasons for not introducing a vaccine against the adult *E*. *granulosus*. Another potential advantage of developing a vaccine for canine echinococcosis is the lower number of dogs compared to livestock population. For example, in China’s Xinjiang province, the dog population has been estimated at 1.5 million, compared to 60 million sheep and goat population in the province. Therefore in terms of logistic and supply fewer animals need to be vaccinated [35]. In a recent study in Kerman, the population of free roaming dogs (FRDs) has been estimated at 6781 dogs i.e. 1.2 dogs per 100 people [36]. According to this dog:human population ratio, total free roaming dog population in Iran can be estimated at 1,020,000 FRDs.

WHO 2021–2030 road map has been delivered global targets and milestones for NTDs control [37]. Echinococcosis is one of the NTDs whose goals have been set for the next ten years based on the control of the disease. In this setting by 2023, 2025 and 2030, intensified control measures for echinococcosis should be implemented in 4, 9, and 17 endemic countries, respectively. The roadmap emphasizes the need for technical requirements, strategy, service delivery, and program capacity to control echinococcosis. Regarding the technical progress, there is a huge gap in the diagnosis of echinococcosis. Planning, governance and program management, monitoring and evaluation, access, and logistics are also crucial in the strategy and service delivery.

Principal steps and critical actions to reach the 2030 goal for echinococcosis include providing baseline data and strengthening integrated national monitoring of CE, developing guidelines for effective prevention and control, as well as strengthening ultrasound diagnosis, effective interventions and ensuring access to albendazole. Implementation of a successful CE control program will require key data including accurate surveillance data for echinococcosis of human, livestock and carnivores. Assessing the impact of control measures and justifying ongoing expenditure of costly control interventions is not possible without suitable surveillance information.

## Conclusion

The results of this study improved our knowledge about the dynamics of canine echinococcosis in southeastern Iran. The findings assist authorities in achieving an appropriate control approach for CE and developing integrated management practices related to endemic zoonoses in the region. With regards to our findings, regular treatment of farm dogs with praziquantel every two months is ideal, however the logistics and cost issues in dog dosing should be considered in a sustainable CE control program. Systematic and in-depth understanding of the epidemiology of human and animal echinococcosis can be valuable for developing effective prevention and control programs according to the WHO road map for neglected tropical diseases 2021–2030.

## Supporting information

S1 Data File GPS-derived coordinates of the sampling sites. (XLSX)

## References

[1] Romig T, Deplazes P, Jenkins D, Giraudoux P, Massolo A, Craig PS, et al. Ecology and Life Cycle Patterns of Echinococcus Species. Adv Parasitol. 2017;95: 213–314. doi: 10.1016/bs.apar.2016.11.002 28131364

[2] Deplazes P, Rinaldi L, Alvarez Rojas CA, Torgerson PR, Harandi MF, Romig T, et al. Global Distribution of Alveolar and Cystic Echinococcosis. Adv Parasitol. 2017;95: 315–493. doi: 10.1016/bs.apar.2016.11.001 28131365

[3] Borhani M, Fathi S, Darabi E, Jalousian F, Simsek S, Ahmed H, et al. Echinococcoses in Iran, Turkey, and Pakistan: Old diseases in the new millennium. Clinical Microbiology Reviews. Am Soc Microbiol; 2021. pp. e00290–20. doi: 10.1128/CMR.00290-20 34076492 PMC8262809

[4] Borhani M, Fathi S, Lahmar S, Ahmed H, Abdulhameed MF, Harandi MF. Cystic echinococcosis in the eastern mediterranean region: Neglected and prevailing! PLoS Negl Trop Dis. 2020;14: 1–3. doi: 10.1371/journal.pntd.0008114 32379760 PMC7205190

[5] Fasihi Harandi M, Budke CM, Rostami S. The Monetary Burden of Cystic Echinococcosis in Iran. PLoS Negl Trop Dis. 2012;6: e1915. doi: 10.1371/journal.pntd.0001915 23209857 PMC3510083

[6] Ansari-Renani HR, Rischkowsky B, Mueller JP, Momen SMS, Moradi S. Nomadic pastoralism in southern Iran. Pastor Res Policy Pract. 2013;3: 1–25.

[7] Eckert J, Gemmell M, Meslin F-X, Pawlowski Z. WHO/OIE Manual on Echinococcosis in Humans and Animals: A Public Health Problem of Global Concern. Veterinary Parasitology. 2002. doi: 10.1016/s0304-4017(01)00631-8

[8] Craig PS, McManus DP, Lightowlers MW, Chabalgoity JA, Garcia HH, Gavidia CM, et al. Prevention and control of cystic echinococcosis. Lancet Infectious Diseases. 2007. pp. 385–394. doi: 10.1016/S1473-3099(07)70134-2 17521591

[9] Craig PS, Hegglin D, Lightowlers MW, Torgerson PR, Wang Q. Echinococcosis: control and prevention. Adv Parasitol. 2017;96: 55–158. 28212791 10.1016/bs.apar.2016.09.002

[10] Buishi IE, Njoroge EM, Bouamra O, Craig PS. Canine echinococcosis in northwest Libya: assessment of coproantigen ELISA, and a survey of infection with analysis of risk-factors. Vet Parasitol. 2005;130: 223–232. doi: 10.1016/j.vetpar.2005.03.004 15905032

[11] Wang YH, Rogan MT, Vuitton DA, Wen H, Bartholomot B, Macpherson CNL, et al. Cystic echinococcosis in semi-nomadic pastoral communities in north-west China. Trans R Soc Trop Med Hyg. 2001;95: 153–158. doi: 10.1016/s0035-9203(01)90142-7 11355546

[12] Lahmar S, Sarciron M-E, Rouiss M, Hammouda A, Youssfi M, Mensi M. Echinococcus granulosus and other intestinal helminths in semi-stray dogs in Tunisia: infection and re-infection rates. Tunis Med. 2008;86: 279–286. 19472727

[13] Moss JE, Chen X, Li T, Qiu J, Wang Q, Giraudoux P, et al. Reinfection studies of canine echinococcosis and role of dogs in transmission of Echinococcus multilocularis in Tibetan communities, Sichuan, China. Parasitology. 2013;140: 1685–1692. doi: 10.1017/S0031182013001200 23985352 PMC3806043

[14] Larrieu E, Costa MT, Cantoni G, Labanchi JL, Bigatti R, Aquino A, et al. Rate of infection and of reinfection by Echinococcus granulosus in rural dogs of the province of Rio Negro, Argentina. Vet Parasitol. 2000;87: 281–286. doi: 10.1016/s0304-4017(99)00180-6 10669098

[15] Cabrera PA, Parietti S, Haran G, Benavidez U, Lloyd S, Perera P, et al. Rates of reinfection with Echinococcus granulosus, Taenia hydatigena, Taenia ovis and other cestodes in a rural dog population in Uruguay. Int J Parasitol. 1996;26: 79–83. doi: 10.1016/0020-7519(95)00082-8 9198601

[16] Wachira TM, Macpherson CNL, Gathuma JM. Hydatid disease in the Turkana District of Kenya, VII: analysis of the infection pressure between definitive and intermediate hosts of Echinococcus granulosus, 1979–1988. Ann Trop Med Parasitol. 1990;84: 361–368. doi: 10.1080/00034983.1990.11812481 2260900

[17] Torgerson PR, Shaikenov BS, Rysmukhambetova AT, Ussenbayev AE, Abdybekova AM, Burtisurnov KK. Modelling the transmission dynamics of Echinococcus granulosus in dogs in rural Kazakhstan. Parasitology. 2003;126: 417–424. doi: 10.1017/s0031182003002932 12793645

[18] Rojas TO, Romero C, Heredia R, Bautista LG, Sheinberg G. Identification of Toxocara spp. eggs in dog hair and associated risk factors. Vet World. 2017;10: 798–802. doi: 10.14202/vetworld.2017.798-802 28831225 PMC5553150

[19] Mesquita JR, Esteves F, Santos C, Mega C, Coelho C, Cruz R, et al. ABC series on diagnostic parasitology part 1: the Willis method. Vet Nurse. 2017;8: 398–402.

[20] Abbasi I, Branzburg A, Campos-Ponce M, Hafez SKA, Raoul F, Craig PS, et al. Copro-diagnosis of Echinococcus granulosus infection in dogs by amplification of a newly identified repeated DNA sequence. Am J Trop Med Hyg. 2003;69: 324–330. 14628952

[21] Mirbadie SR, Kamyabi H, Mohammadi MA, Shamsaddini S, Harandi MF. Copro-PCR prevalence of Echinococcus granulosus infection in dogs in Kerman, south-eastern Iran. J Helminthol. 2018;92: 17–21. doi: 10.1017/S0022149X17000074 28132657

[22] Rue H, Riebler A, Sørbye SH, Illian JB, Simpson DP, Lindgren FK. Bayesian computing with INLA: a review. Annu Rev Stat Its Appl. 2017;4: 395–421.

[23] Moraga P. Geospatial health data: Modeling and visualization with R-INLA and shiny. CRC Press; 2019.

[24] Leaflet—an open-source JavaScript library for interactive maps. 2023 [cited 25 Aug 2023]. Available from: https://leafletjs.com/

[25] Craig PS, Rogan MT, Campos-Ponce M. Echinococcosis: disease, detection and transmission. Parasitology. 2003;127: S5. 15027602

[26] Kumaratilake LM, Thompson RCA, Dunsmore JD. Comparative strobilar development of Echinococcus granulosus of sheep origin from different geographical areas of Australia in vivo and in vitro. Int J Parasitol. 1983;13: 151–156. doi: 10.1016/0020-7519(83)90005-x 6853016

[27] Amarir FE, Saadi A, Marcotty T, Rhalem A, Oukessou M, Sahibi H, et al. Cystic echinococcosis in three locations in the Middle Atlas, Morocco: Estimation of the infection rate in the dog reservoir. Vector-Borne Zoonotic Dis. 2020;20: 436–443. doi: 10.1089/vbz.2019.2538 32077790

[28] Šarkūnas M, Vienažindienė Ž, Rojas CAA, Radziulis K, Deplazes P. Praziquantel treatment of dogs for four consecutive years decreased the transmission of Echinococcus intermedius G7 to pigs in villages in Lithuania. Food waterborne Parasitol. 2019;15: e00043. doi: 10.1016/j.fawpar.2019.e00043 32095615 PMC7033992

[29] Lashkarizadeh MR, Hooshmand N, Nasibi S, Mohammadi MA, Shamsaddini S, Kamyabi H, et al. Genetic profile of hydatid cysts in patients with multi-organ involvement: mixed infections by different strains. Vector-Borne Zoonotic Dis. 2019;19: 724–730. doi: 10.1089/vbz.2018.2427 30920351

[30] Thompson RCA. Biology and Systematics of Echinococcus. Adv Parasitol. 2017;95: 65–109. doi: 10.1016/bs.apar.2016.07.001 28131366

[31] Thompson RCA, Lymbery AJ, Constantine CC. Variation in Echinococcus: towards a taxonomic revision of the genus. Adv Parasitol. 1995;35: 145–175. doi: 10.1016/s0065-308x(08)60071-8 7709852

[32] Leblebicioglu H, Sunbul M, Memish ZA, Al-Tawfiq JA, Bodur H, Ozkul A, et al. Consensus report: Preventive measures for Crimean-Congo Hemorrhagic Fever during Eid-al-Adha festival. Int J Infect Dis. 2015;38: 9–15. doi: 10.1016/j.ijid.2015.06.029 26183413

[33] Ebrahimipour M, Budke CM, Harandi MF. Control of Cystic Echinococcosis in Iran: Where Do We Stand? Trends in Parasitology. Elsevier; 2020. pp. 578–581. doi: 10.1016/j.pt.2020.04.007 32402838

[34] Sharifi I, Daneshvar H, Ziaali N, Nikian Y, Ebrahimi AM, Keshavarz H, et al. Evaluation of a control program on hydatid cyst in the city of Kerman. J Kerman Univ Med Sci. 1996;3: 168–174.

[35] Zhang W, McManus DP. Vaccination of dogs against Echinococcus granulosus: a means to control hydatid disease? Trends in Parasitology. 2008. pp. 419–424. doi: 10.1016/j.pt.2008.05.008 18678528

[36] Shamsaddini S, Ahmadi Gohari M, Kamyabi H, Nasibi S, Derakhshani A, Mohammadi MA, et al. Dynamic modeling of female neutering interventions for free-roaming dog population management in an urban setting of southeastern Iran. Sci Rep. 2022;12: 4781. doi: 10.1038/s41598-022-08697-w 35314736 PMC8938497

[37] WHO. Ending the neglect to attain the Sustainable Development Goals: A road map for neglected tropical diseases 2021–2030. In: WHO [Internet]. 2020 [cited 1 Dec 2020]. Available from: https://www.who.int/neglected_diseases/resources/who-ucn-ntd-2020.01/en/

